# Syndrome of Inappropriate Antidiuretic Hormone Secretion and Lead Toxicity in a Child With Sickle Cell Disease and Pica

**DOI:** 10.7759/cureus.16813

**Published:** 2021-08-01

**Authors:** Akshay Gupta, Alexandra Amaducci, Andrew Koons, John D Lindmark, Gillian A Beauchamp

**Affiliations:** 1 Department of Emergency and Hospital Medicine, Lehigh Valley Health Network/University of South Florida Morsani College of Medicine, Allentown, USA; 2 Division of Medical Toxicology, Department of Emergency and Hospital Medicine, Lehigh Valley Health Network/University of South Florida Morsani College of Medicine, Allentown, USA; 3 Department of Pediatric Critical Care, Lehigh Valley Health Network/University of South Florida Morsani College of Medicine, Allentown, USA

**Keywords:** sickle cell, lead toxicity, pediatric, anemia, toxic, case report

## Abstract

We describe the presentation and management of a three-year-old child with a history of pica, vitamin D deficiency, and sickle cell disease, who was admitted for pyelonephritis, and found to have elevated blood lead level (BLL) of 103.7 µg/dL, and who subsequently developed altered mental status and syndrome of inappropriate antidiuretic hormone secretion (SIADH). In consultation with Medical Toxicology, the patient was chelated with calcium disodium edetate (EDTA) and British Anti Lewisite (BAL). The patient’s hyponatremia was managed with hypertonic saline infusion. The patient’s encephalopathy improved throughout her hospital course, and she was discharged on hospital day 8. Following five days of EDTA and three days of BAL injections, her repeat BLL was 15.3 µg/dL. SIADH has been associated with severe lead poisoning and may be more likely to occur in high risk patients such as individuals with sickle cell anemia, particularly where medications are used that may cause iatrogenic hyponatremia.

## Introduction

According to The American Academy of Pediatrics, impaired cognition may be associated with a blood lead level (BLL) greater than 5 µg/dL [1]. Significantly elevated BLL (>100 µg/dL) can result in emesis, encephalopathy, and death. Both lead toxicity and sickle cell disease-induced vaso-occlusive crisis can present with nausea, vomiting, constipation, abdominal pain, arthralgias, and anemia potentially leading to challenges in diagnosis and treatment.

Hyponatremia has been reported in association with both sickle cell disease [2] and lead toxicity [3,4]. Further, hyponatremia has additionally been reported in the context of the syndrome of inappropriate antidiuretic hormone section (SIADH) in association with lead toxicity [5,6].

We report the case of a three-year-old female with type SS sickle cell anemia who developed encephalopathy in the context of lead toxicity and SIADH. We discuss the potentially multifactorial nature of the patient’s SIADH including the potential contribution of sickle anemia, pica, lead toxicity, and the use of medications associated with the development of iatrogenic hyponatremia.

This article was previously presented as a meeting abstract at the 2020 North American Congress of Clinical Toxicology (NACCT).

## Case presentation

A three-year-old girl arrived at a community hospital emergency department (ED) with vomiting and abdominal pain. Two days prior to presentation, the patient developed abdominal pain and generalized malaise. She had an underlying medical history of vitamin D deficiency, functional asplenia, and type SS sickle cell anemia with hemoglobin typically ranging between 7 and 8 g/dL. Her daily medications included hydroxyurea, folic acid, vitamin D, and amoxicillin. Based on previous lead screening, the patient had known mild lead elevations with the highest reported at 24 µg/dL approximately 17 months prior to her presentation.

The ED medical team diagnosed the patient with pyelonephritis. She received intravenous (IV) ceftriaxone and was admitted to the hospital. A whole BLL was sent due to her chronically elevated outpatient lead concentrations. Admission laboratory studies demonstrated sodium of 141 mmol/L. She was maintained on an IV infusion of 5% dextrose in normal saline (D5NS) due to poor oral intake. On hospital day (HD) 1, she continued to complain of abdominal pain. An abdominal radiograph showed nonspecific gaseous distention. At that point, an enema and polyethylene glycol 3350 were administered. These interventions mildly improved her stool output and oral intake. On HD 2, Escherichia coli was detected in her urine culture. After having received a brief course of IV ceftriaxone, ciprofloxacin was initiated based on antibiotic sensitivities. On HD 3, her repeat BLL resulted at 103.7 µg/dL prompting transfer to our toxicology center for further treatment.

When the patient arrived at our facility, she had the following vital signs: heart rate 120 beats per minute, blood pressure 124/73, respiratory rate 30/minute, oxygen saturation 100%, temperature 99.5 degrees Fahrenheit. Her repeat bloodwork demonstrated: sodium 128 mmol/L, potassium 4.6 mmol/L, chloride 93 mmol/L, carbon dioxide 23 mmol/L, calcium 10.4 mg/dL, glucose 109 mg/dL, and BLL 110.2 µg/dL. The medical toxicology service was consulted, and the patient was chelated with British Anti Lewisite (BAL) followed by calcium disodium edetate (EDTA). She was initially continued on ciprofloxacin, D5NS maintenance fluids, and her outpatient medications. During the evening of HD 4, the patient developed episodic irritability and confusion. On the morning of HD 5, in the context of worsening intermittent confusion, the repeat sodium level was 115 mmol/L. Her level of care was upgraded to pediatric intensive care where a hypertonic saline bolus and infusion were initiated. Laboratory testing performed just prior to initiation of hypertonic saline revealed a plasma sodium concentration of 112 mmol/L, serum osmolality of 253 mOsm/kg, urine sodium concentration of 77 mmol/L, and urine osmolality of 211 mOsm/kg. Ciprofloxacin was discontinued and cefdinir was initiated. She had received four total doses of ciprofloxacin. The intensive care team reduced her maintenance fluids to half of the recommended volume by weight. Her sodium improved to 125 mmol/L within nine hours. At that point, the hypertonic saline infusion was discontinued. Her sodium continued to rise overnight, and by the morning of HD 6, her sodium was 138 mmol/L. Given that the initial infusions of EDTA were prepared in 500 cc of 5% dextrose in water (D5W), on HD 6, we concentrated it into 250 cc of normal saline solution to limit any unnecessary free water. The patient’s sodium remained within normal range and her encephalopathy continued to improve until her discharge on HD 8 when her BLL after five days of EDTA and three days of BAL was 15.3 µg/dL.

The child was seen in our toxicology clinic approximately one month later, at which point her mother reported that she was asymptomatic. She was meeting her developmental milestones. Her BLL at that time was 17.9 µg/dL.

## Discussion

This case of a three-year-old female with type SS sickle cell anemia who developed encephalopathy in the context of lead toxicity and SIADH, illustrates the potentially multifactorial nature of SIADH including a history of both sickle anemia and pica, lead toxicity, and the use of medications associated with the development of iatrogenic hyponatremia.

This patient had an elevated BLL in the context of frequent paint chip ingestion due to pica. In general, BLL has decreased dramatically in the US over the past four decades since the elimination of lead from gasoline paints and other consumer products, but many children still live in housing with deteriorated lead-based paint [1]. The American Academy of Pediatrics (AAP) reports that BLL even below 5 µg/dL impair cognition and therefore there is no safe level of lead in blood [1]. Patients that have very high BLL (>100 µg/dL) can have significant features such as protracted vomiting and encephalopathy and even death. Elevated BLL is a risk factor for diminished intellectual and academic abilities, higher rates of neurobehavioral disorders such as hyperactivity and attention deficits, and lower birth weights in children. No effective treatments can completely reduce the permanent developmental effects of lead toxicity [1].

Hyponatremia in the setting of the sickle cell has been well-described. In a chart review of 46 patients with sickle cell disease, hyponatremia was observed in 52% of cases [2]. The authors concluded that this finding was likely secondary to a renally mediated water and salt-wasting state. However, we note that many patients in this report did indeed have urine sodium and osmolality measurements consistent with SIADH.

Additionally, hyponatremia in the setting of lead toxicity has been described, but urine sodium and osmolality measurements were not included in either of these reports, so no determination of SIADH could be made [3,4]. The association of SIADH with lead toxicity was first proposed by Suarez et al. in 1992 [5]. They described a five-year-old patient with sickle cell anemia who had a BLL of 135 µg/dL. Her sodium concentration declined from 145 mEqL to 114 mEq/L over four HDs, with urine osmolality and sodium concentration consistent with SIADH. The patient had rapid improvement with fluid restriction, hypertonic saline, and chelation. Issaivanan et al reported a similar case of a three-year-old child with sickle cell anemia who had a BLL of 227 µg/dl, and an initial sodium concentration of 132 meq/L that decreased to 119 meq/L by day 3, also with urine sodium and osmolality consistent with SIADH [6].

 In a retrospective review of 480 patients with sickle cell disease, Ivascu et al. reported pica behavior in 14.8%-23.3% among those age 10-19 years old [7]. Issaivanan et al. reported a prevalence of pica to be 76% in younger children [6]. Jung et al. noted multiple additional risk factors for young children to develop lead poisoning including lower socioeconomic status, black race, and Hispanic ethnicity [8]. In addition, an incomplete blood-brain barrier allows for increased lead penetration to the developing brain [8]. Patients with sickle cell anemia more commonly have iron deficiency anemia, which leads to increased gastrointestinal absorption of lead [8,9].

The patient we describe here developed SIADH in the setting of both lead toxicity and fluoroquinolone administration. An association between fluoroquinolones and SIADH has been reported previously [10-12]. It is difficult to establish a causal association between fluoroquinolones and SIADH in these case reports given the use of other medications or other underlying disease states that may also be associated with SIADH or hyponatremia. In vitro models have suggested fluoroquinolones are agonists at the N-methyl-D-aspartate (NMDA) receptor [13]. Antidiuretic hormone (ADH) neurosecretory cells are primarily located in the periventricular nucleus (PVN) and supraoptic nucleus (SON) of the hypothalamus, and glutamate has been identified as an important neurotransmitter governing the release of ADH via both ionotropic non-NMDA and NMDA receptors [14,15]. In vitro studies suggested that both non-NMDA and NMDA receptor activation were necessary for stimulation of ADH release (Morsette) [15]. However, more recent in vivo evidence reveals that ADH release in both the SON and PVN results from primary activation of non-NMDA glutamate receptors [14]. Interestingly, PVN ADH release appears to be dependent on not only non-NMDA receptor activation but also NMDA receptor inhibition [14]. Although the neuroregulation of ADH likely involves more than glutamate receptor interactions, these receptors may be pertinent in the setting of both fluoroquinolone use and lead toxicity.

## Conclusions

This case of a three-year-old female with sickle cell anemia and pica who developed SIADH in the context of concomitant lead toxicity and a urinary tract infection treated with a fluoroquinolone demonstrates that hyponatremia may be multifactorial. SIADH may be associated with severe lead poisoning and may be more likely among high-risk patients with sickle cell anemia, particularly where medications associated with hyponatremia are involved. Where multiple risk factors for SIADH are present, evaluation for this disease process should be undertaken in patients who develop hyponatremia. Lead toxicity should be considered in patients with risk factors such as sickle cell anemia, pica, lower socioeconomic status, and non-specific presenting features such as gastrointestinal distress.
